# Association between migraine and Alzheimer’s disease: a nationwide cohort study

**DOI:** 10.3389/fnagi.2023.1196185

**Published:** 2023-05-25

**Authors:** Jaeho Kim, Woo Seok Ha, Sang Hyun Park, Kyungdo Han, Min Seok Baek

**Affiliations:** ^1^Department of Neurology, Dongtan Sacred Heart Hospital, Hallym University College of Medicine, Hwaseong-si, Republic of Korea; ^2^Department of Neurology, Gangwon-do Wonju Medical Center, Wonju, Republic of Korea; ^3^Department of Neurology, Severance Hospital, Yonsei University College of Medicine, Seoul, Republic of Korea; ^4^Department of Statistics and Actuarial Science, Soongsil University, Seoul, Republic of Korea; ^5^Department of Neurology, Wonju Severance Christian Hospital, Yonsei University Wonju College of Medicine, Wonju, Republic of Korea; ^6^Research Institute of Metabolism and Inflammation, Yonsei University Wonju College of Medicine, Wonju, Republic of Korea

**Keywords:** migraine, dementia, Alzheimer’s disease, headache, cohort studies

## Abstract

**Background and objective:**

Migraine is a common chronic neurological disease characterized by pulsating headaches, photophobia, phonophobia, nausea, and vomiting. The prevalence of dementia in individuals aged over 65 years in Korea is more than 10%, and Alzheimer’s disease (AD) dementia accounts for most cases. Although these two neurological diseases account for a large portion of the medical burden in Korea, few studies have examined the relationship between the two diseases. Therefore, this study investigated the incidence and risk of AD in patients with migraines.

**Methods:**

We retrospectively collected nationwide data from a national health insurance claims database governed by Korea’s National Health Insurance Service. Among Koreans in the 2009 record, patients with migraine were identified according to the International Classification of Diseases, 10th revision (ICD-10) code G43. First, we screened the database for participants aged over 40 years. Individuals diagnosed with migraine at least twice over more than 3 months in a year were considered to have chronic migraine in this study. Further, all participants with an AD diagnosis (ICD-10 code: Alzheimer’s disease F00, G30) were investigated for AD dementia development. The primary endpoint was AD development.

**Results:**

The overall incidence of AD dementia was higher in individuals with a history of migraine than in those with no migraine history (8.0 per 1,000 person-years vs. 4.1 per 1,000 person-years). The risk of AD dementia was higher in individuals diagnosed with migraine (hazard ratio = 1.37 [95% confidence interval, 1.35–1.39]) than in the control group after adjustments for age and sex. Individuals with chronic migraine had a higher incidence of AD dementia than those with episodic migraine. Younger age (<65 years old) was associated with an increased risk of AD dementia compared to older age (≥65  years old). Higher body mass index (BMI) (≥25 kg/m^2^) was also associated with an increased risk of AD dementia compared to lower BMI (<25 kg/m^2^) (*p* < 0.001).

**Conclusion:**

Our results suggest that individuals with a migraine history are more susceptible to AD than those without a migraine history. Additionally, these associations were more significant in younger and obese individuals with migraine than in individuals without migraine.

## 1. Introduction

Migraine is a neurological disorder that causes symptoms such as headache, abdominal discomfort, vomiting, and visual impairment, affecting more than 10 billion people worldwide (Amiri et al., 2021). Alzheimer’s disease (AD) is the most common cause of dementia, with more than 30 million people affected worldwide (Gustavsson et al., 2023). Female sex, old age, hypertension, diabetes, obesity, dyslipidemia, stress, and hormonal imbalance were considered risk factors for migraine (Giannini et al., 2012; Amiri et al., 2021). Old age, female sex, hypertension, diabetes, dyslipidemia, and obesity are well-known risk factors for AD (Leibson et al., 1997; Qiu et al., 2003; Reitz, 2013; Lloret et al., 2019; Ding et al., 2020). Although these two neurological diseases account for a large portion of the medical burden worldwide, few studies have examined their relationship.

Previous studies showed inconsistent results regarding the association between migraine and AD (Morton et al., 2019; George et al., 2020; Islamoska et al., 2020). However, more recent large-scale studies have reported that patients with migraine have a higher incidence of AD than those without migraine (Kostev et al., 2019; Lee et al., 2021). Regarding the association between dementia and migraine, several studies have suggested the existence of a relationship between white matter damage, depression, chronic pain, and stress (Jensen, 2003; Wang et al., 2018); however, there are few studies on the association according to common risk factors for migraine and dementia, especially AD.

Extensive research into the association between migraine and dementia could improve the diagnosis and treatment of these disorders that significantly affect patients’ quality of life. In the present study, we aimed to investigate the association between migraine and the incidence of AD dementia according to various risk factors.

## 2. Methods

### 2.1. Participants

We retrospectively collected nationwide data from a national health insurance claims database governed by Korea’s National Health Insurance Service (NHIS). Among 10,628,070 Koreans who participated in the national health screening program in 2009, individuals aged ≥40 years were eligible for inclusion in this study. We investigated the medical records of the eligible participants as obtained from the NHIS database during the period spanning from 2002 to 2019. The date of participation in the 2009 national health screening program was defined as the start date of the follow-up. The patients with migraine were identified according to the International Classification of Diseases, 10th revision (ICD-10) code G43. Patients diagnosed with migraine at least twice over more than 3 months in a year were considered to have chronic migraine in this study.

### 2.2. Outcome

All participants were investigated for AD development based on a positive AD diagnosis (ICD-10 code Alzheimer’s disease F00, G30) and their prescription records for anti-dementia medication. The primary endpoint was AD development. Participants with a previous AD diagnosis were excluded from the study. Participants were also excluded if they developed AD or died within 12 months of enrollment. The wash-out period of 12 months was used to minimize the possibility of reverse-causality. The study flowchart is presented in Supplementary Figure S1.

### 2.3. Statistical analyses

To investigate whether migraine affects the risk of AD, we calculated the hazard ratios (HR) and the associated 95% confidence intervals (CI) using the Cox proportional hazards model. We used three progressively adjusted models. Model 1 involved a crude analysis without any adjustment. Model 2 was adjusted for age and sex. In model 3, we adjusted age, sex, comorbidities, lifestyle factors (smoking, drinking, and physical exercise), eGFR, and BMI. Multivariate Cox regression analysis was used in subgroup analyses to assess the impact of underlying comorbidities or demographic characteristics on AD risk in individuals with migraine. All analyses were performed using the SAS statistical software (version 9.2; SAS Institute, Cary, NC, United States).

## 3. Results

### 3.1. Demographics of participants

The demographic characteristics of the study participants are shown in Table 1. Female patients had a higher predominance of migraine than male patients (72.3% vs. 46.6%, *p* < 0.001). Hypertension, diabetes, dyslipidemia, myocardial infarction, stroke, and congestive heart failure were significantly more predominant in patients with migraine than the participants without migraine (*p* < 0.001). Patients with migraine comprised a higher percentage of never smokers and non-drinkers than the participants without migraine (*p* < 0.001).

**Table 1:** Demographic characteristics.

	Number (%)		
	No migraine	Migraine	*p* value
No.	5,863,348	212,836	
Age, years	54.0 ± 10.3	56.5 ± 10.9	<0.001
Female sex	2,734,541 (46.6)	153,766 (72.3)	<0.001
*Comorbidities*			
Hypertension	1,996,596 (34.1)	90,278 (42.4)	<0.001
Diabetes	680,214 (11.6)	23,679 (11.1)	<0.001
Dyslipidemia	1,238,819 (21.1)	59,135 (27.8)	<0.001
Myocardial infarction	28,983 (0.5)	1751 (0.8)	<0.001
Stroke	106,465 (1.8)	12,216 (5.7)	<0.001
Congestive heart failure	41,904 (0.7)	3,238 (1.5)	<0.001
BMI, kg/m^2^	24.0 ± 3.0	24.0 ± 3.1	<0.001
Waist circumferences, cm	81.3 ± 8.6	80.5 ± 8.7	<0.001
eGFR, mL/min/1.73 m^2^	85.0 ± 38.2	83.7 ± 32.0	<0.001
Smoking			<0.001
Never smoker	3,588,441 (61.2)	169,257 (79.5)	
Ex-smoker	961,144 (16.4)	20,548 (9.7)	
Current Smoker	1,313,763 (22.4)	23,031 (10.8)	
Alcohol consumption			<0.001
Non-drinker	3,314,642 (56.5)	157,915 (74.2)	
Moderate (<30 g/day)	2,091,280 (35.7)	47,406 (22.3)	
Heavy	457,426 (7.8)	7,515 (3.5)	

Continuous variables are expressed as means ± standard deviations. Categorical variables are expressed as frequencies (%). BMI, body mass index (calculated as weight in kilograms divided by height in meters squared); eGFR, estimated glomerular filtration rate.

### 3.2. Impact of migraine on the incidence of AD

The overall incidence of AD in participants with no migraine history was 3.7% (214,414/5,863,348), whereas that in those with a migraine history was 7.1% (15,111/2,12,836) (Table 2), suggesting that migraine was related to the incidence of AD.

**Table 2:** Cox proportional hazard regression analysis of the risk of Alzheimer’s disease in participants with different types of migraine.

					HR (95% CI)		
	No.	Positive AD diagnosis	Person-years	Incidence per 1,000 persons, y	Model 1	Model 2	Model 3
No migraine history	5,863,348	214,414	52640741.1	4.07316	1 (Ref.)	1 (Ref.)	1 (Ref.)
Migraine	212,836	15,111	1877684.6	8.04768	1.974 (1.942,2.007)	1.371 (1.349,1.394)	1.323 (1.301,1.345)
Migraine without aura	203,517	14,535	1794787	8.09845	1.987 (1.954,2.021)	1.371 (1.348,1.394)	1.323 (1.301,1.345)
Migraine with aura	9,319	576	82897.5	6.94834	1.699 (1.566,1.844)	1.375 (1.267,1.492)	1.325 (1.221,1.438)
Episodic migraine	154,585	10,312	1368354.8	7.53606	1.847 (1.811,1.884)	1.305 (1.279,1.331)	1.261 (1.236,1.287)
Chronic migraine	58,251	4,799	509329.7	9.42219	2.321 (2.255,2.388)	1.541 (1.498,1.586)	1.479 (1.437,1.522)

Model 1: Unadjusted. Model 2: Adjusted for age and sex. Model 3: Adjusted for age, sex, comorbidities (hypertension, diabetes, dyslipidemia, myocardial infarction, congestive heart failure, and stroke), eGFR, BMI, and lifestyle (smoking status, drinking status, and regular exercise). AD, Alzheimer’s disease; HR, hazard ratio; CI, confidence interval.

Cox proportional hazard regression analysis showed that patients with migraine developed AD with a 1.32 HR (95% CI, 1.30–1.35) compared to the participants with no migraine history after adjustments for age, sex, comorbidities, and lifestyle (Table 2). In the subgroup analysis, we observed that patients with chronic migraine had a higher HR of AD development than those with episodic migraine (HR = 1.48 [95% CI, 1.44–1.52] vs. HR = 1.26 [95% CI, 1.27–1.29]).

**Figure 1:**
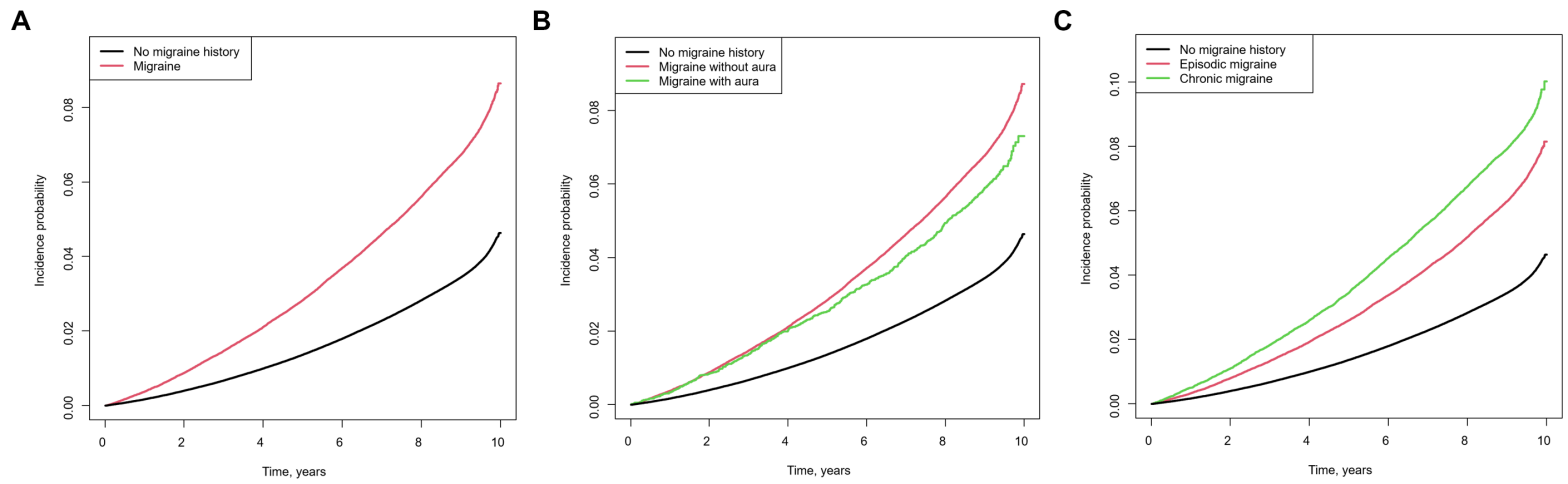
Kaplan–Meier curves of the cumulative incidence of Alzheimer’s disease in individuals with migraine Migraine versus no migraine history **(A)**, chronic migraine versus episodic migraine versus no migraine history **(B)**, migraine with aura versus migraine without aura versus no migraine history **(C)**.

Figure 1 shows the Kaplan–Meier curves of the incidence of AD. The cumulative incidence of AD was significantly higher in participants with versus without a migraine history (Figure 1A). In the subgroup analysis, the migraine without aura group showed a higher incidence of AD than the migraine with aura group (Figure 1B). Furthermore, patients with chronic migraine had a higher cumulative incidence of AD than those with episodic migraine (Figure 1C).

### 3.3. Effects of risk factors of AD in individuals with migraine

We evaluated whether AD risk factors had differential effects between participants with migraine and controls. In the younger age group (age < 65 years), participants with migraine had a higher incidence of AD (HR = 1.58 [95% CI, 1.52–1.64]) than those without migraine. This was higher than that in the older age group (aged ≥65 years) compared to those without migraine (HR = 1.27 [95% CI, 1.24–1.30]; *P* for interaction <0.001) after adjustments for covariates (Table 3).

**Table 3:** Multivariate Cox proportional hazards regression analysis of the risk of Alzheimer’s disease in participants with migraine.

		Group	No	Positive AD diagnosis	Person-years	Incidence per 1,000 persons, y	Model 3, HR (95% CI)	*p* value
**Age**	Age < 65 years	Control	4,821,001	42,556	44383171.9	0.9588	1 (Ref.)	<0.001
		Migraine	158,240	2,680	1459465.08	1.8363	1.576 (1.516,1.639)	
	Age ≥ 65 years	Control	1,042,347	171,858	8257569.2	20.8122	1 (Ref.)	
		Migraine	54,596	12,431	418219.5	29.7236	1.267 (1.244,1.29)	
**Sex**	Men	Control	3,128,807	89,299	27924657.7	3.19785	1 (Ref.)	0.381
		Migraine	59,070	3,425	512588.72	6.68177	1.305 (1.262,1.351)	
	Women	Control	2,734,541	125,115	24716083.4	5.06209	1 (Ref.)	
		Migraine	153,766	11,686	1365095.86	8.56057	1.328 (1.303,1.354)	
**BMI**	BMI < 25 kg/m^2^	Control	3,822,407	142,246	34228948.2	4.15572	1 (Ref.)	<0.001
		Migraine	137,202	9,645	1207476.61	7.98773	1.289 (1.262,1.316)	
	BMI ≥25 kg/m^2^	Control	2,040,941	72,168	18411792.9	3.91966	1 (Ref.)	
		Migraine	75,634	5,466	670207.96	8.15568	1.391 (1.353,1.43)	
**Diabetes**	No	Control	5,183,134	165,669	46823813.1	3.5381	1 (Ref.)	0.371
		Migraine	189,157	11,851	1683329.43	7.0402	1.328 (1.304,1.353)	
	Yes	Control	680,214	48,745	5816927.99	8.3799	1 (Ref.)	
		Migraine	23,679	3,260	194355.15	16.7734	1.304 (1.259,1.351)	
**Hypertension**	No	Control	3,866,752	84,358	35259722.3	2.3925	1 (Ref.)	0.200
		Migraine	122,558	5,092	1109718.32	4.5886	1.343 (1.306,1.382)	
	Yes	Control	1,996,596	130,056	17381018.7	7.4826	1 (Ref.)	
		Migraine	90,278	10,019	767966.26	13.0461	1.313 (1.286,1.34)	
**Dyslipidemia**	No	Control	4,624,529	151,982	41617455.2	3.6519	1 (Ref.)	0.040
		Migraine	153,701	9,653	1363593.29	7.0791	1.306 (1.279,1.333)	
	Yes	Control	1,238,819	62,432	11023285.9	5.6636	1 (Ref.)	
		Migraine	59,135	5,458	514091.29	10.6168	1.354 (1.317,1.392)	
**Smoking**	No	Control	4,549,585	184,297	40910768.7	4.50485	1 (Ref.)	0.289
		Migraine	189,805	13,952	1676906.83	8.32008	1.327 (1.304,1.35)	
	Yes	Control	1,313,763	30,117	11729972.4	2.56753	1 (Ref.)	
		Migraine	23,031	1,159	200777.75	5.77255	1.283 (1.21,1.361)	

AD, Alzheimer’s disease; HR, hazard ratio; CI, confidence interval; BMI, body mass index.

Additionally, in the obese group (BMI ≥25 kg/m^2^), participants with migraine showed a higher incidence of AD than those without migraine (HR = 1.39 [95% CI, 1.35–1.43]). The incidence of AD was also higher in the non-obese group (BMI < 25 kg/m^2^) than in the controls (HR = 1.29 [95% CI, 1.26–1.32]; *P* for interaction <0.001) (Table 3).

**Figure 2:**
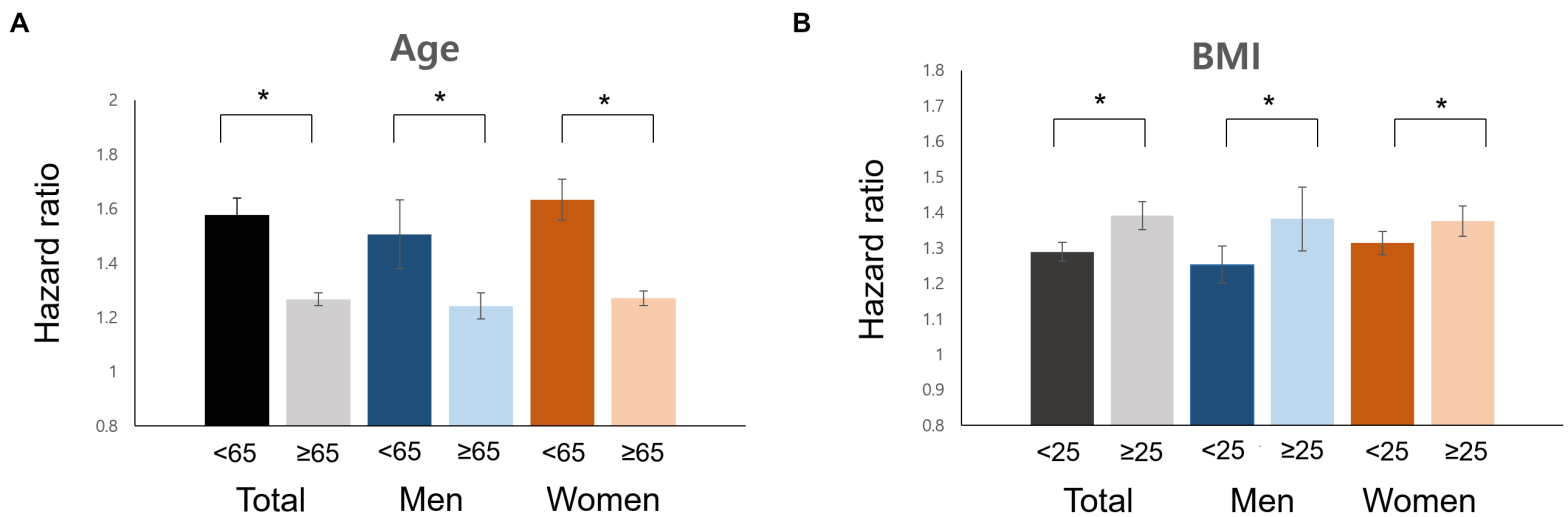
Impacts of risk factors for Alzheimer’s disease in men and women with migraine. Age **(A)**, BMI **(B)**, hazard ratio. BMI, body mass index.

In the subgroup analysis, the interaction of age and obesity with AD incidence was consistent in both men and women (Figure 2, *p* < 0.05). There were no significant differences in the effects of sex, diabetes, hypertension, dyslipidemia, and current smoking status for any interaction of migraine with the incidence of AD.

## 4. Discussion

The present study evaluated the association between migraine and AD in a large-scale Korean nationwide population-based cohort. We found a higher incidence of AD in individuals with migraine than in those without migraine. We also found a higher AD incidence in individuals with chronic migraine than in those with episodic migraines. Lastly, we showed that younger age and higher BMI increased the risk of AD in patients with migraine.

Our first major finding was that the overall incidence of AD among individuals with migraine was higher than that in individuals with no migraine history. This result is consistent with those of previous studies that suggest that migraine is associated with an increased risk of dementia (Wang et al., 2018; Lee et al., 2021; Gu et al., 2022). We analyzed specific personal, medical, and behavioral data, including BMI, smoking history, and alcohol consumption. The prevalence rate of migraine and AD may vary depending on race-related differences (Stewart et al., 1996; Niu et al., 2017; Park et al., 2019; Kim et al., 2022), while the association between these two conditions could be influenced by disparities in lifestyle habits and socioeconomic status. Recently, Gu et al. (2022) revealed that migraine was associated with an increased risk of dementia, suggesting a significant association with cognitive decline in several country-based cohorts. Although several studies have recently reported an association between migraine and dementia, the pathomechanisms underlying the occurrence of cognitive decline in patients with migraine remain unclear. Migraine is a painful condition. Many common structures exist in the pain pathway and memory processing circuits, such as the thalamus, insula, anterior cingulate, hippocampus, and temporal cortex (Apkarian et al., 2005; Svoboda et al., 2006; Liu et al., 2018). Chronic repetitive pain can cause vulnerability of these brain structures and weaken brain function, resulting in memory deterioration.

Our second major finding was that individuals with chronic migraine showed a higher rate of AD development than individuals with episodic migraine. Due to the limited evaluation of the dataset, we set the criterion for a chronic headache diagnosis as at least two migraine diagnoses over more than 3 months in a year, contrary to the diagnostic criteria for chronic migraine. Chronic stress activates the hypothalamic–pituitary–adrenocortical (HPA) axis, resulting in glucocorticoid release and HPA axis dysregulation (Herman et al., 2016). Recent studies have shown that growing evidence supports the association between HPA axis dysregulation and amyloidosis and synaptic plasticity disruption related to AD progression (Chi et al., 2014; Saeedi and Rashidy-Pour, 2021). We conjectured that individuals with chronic migraine are exposed to repetitive and extensive chronic stress, and this can accumulate, resulting in a higher incidence of AD development.

Additionally, in the multivariable analysis, we found that younger and obese individuals with migraine had a higher AD incidence than controls. There were no significant differences in the effect of other AD risk factors such as sex, diabetes, hypertension, dyslipidemia, and current smoking status for any interaction with migraine on the rate of AD development. It is known that vascular risk factors such as diabetes, hypertension, dyslipidemia, and current smoking are associated with AD (Leibson et al., 1997; Reitz, 2013; Chi et al., 2014; Durazzo et al., 2014; Ding et al., 2020). Gomez et al. demonstrated that vascular risk factors could activate brain amyloid-ß accumulation by reducing amyloid-ß clearance and increasing oxidative, inflammatory stress response (Gomez et al., 2018). A previous study showed that obesity could contribute to cognitive dysfunction by activating systemic inflammatory processes (Nguyen et al., 2014). The results of this study indicate that obese individuals with migraine may be more susceptible to AD than those without a migraine history. Based on our results, we assume that the greater the AD development in younger patients with migraine, the greater the long-term or earlier impact of these stresses on brain structure and function related to memory processes.

### 4.1. Strengths and limitations

Our study has a few limitations. First, we collected retrospective data from a large health insurance claims databases, which can have incomplete or inaccurate coding, and the lack of detailed clinical information on the participants. Second, we used the ICD-10 diagnostic code to diagnose migraine and AD, did not use specific medical records for migraine, and AD was not confirmed by amyloid or tau biomarkers. Third, we did not evaluate the effect of migraine-specific medications on cognitive function and dementia risk such as triptans. Fourth, our study does not establish a causal relationship between the migraine and AD Further studies are needed to clarify the underlying mechanisms and potential confounding factors. Finally, it is necessary to investigate the relationship between various risk factors and diseases through follow-up studies. Despite these limitations, using a large nationwide population-based dataset with longitudinal observation seems valuable, providing a homogenous sample.

## 5. Conclusion

Our results suggest that individuals with a migraine history are more susceptible to AD than individuals without a migraine history. Additionally, these associations were more significant in individuals with chronic migraine and obese and younger individuals with migraine than in individuals without migraine. Our findings will encourage clinicians to consider individuals with migraine should be followed-up and corrected for the risk factors for AD dementia. Further studies are warranted to evaluate whether these risk factors influence AD exacerbation.

## Data availability statement

The original contributions presented in the study are included in the article/Supplementary material, further inquiries can be directed to the corresponding author.

## Ethics statement

Ethical review and approval was not required for the study on human participants in accordance with the local legislation and institutional requirements. Written informed consent for participation was not required for this study in accordance with the national legislation and the institutional requirements.

## Author contributions

JK: drafting or revising the manuscript content, including medical science writing, study concept or design, Analysis or interpretation of data. WH: study conceptualization or drafting the study design. SP: study conceptualization or drafting the study design, analysis or interpretation of data. KH: study conceptualization or drafting the study design, major role in data acquisition, and analysis or interpretation of data. MB: drafting or revision of the manuscript, study conceptualization or drafting the study design, analysis or interpretation of data.

All authors contributed to the article and approved the submitted version.

## Funding

This research was supported by grants from the National Research Foundation (NRF) funded by the Ministry of Education (NRF2022R1C1C1012535, NRF-2022R1C1C1010435), the Technology Development Program (S3030742) funded by the Ministry of SMEs and Startups (MSS, Korea), and the Technology Innovation Program (20018182) funded by the Ministry of Trade, Industry & Energy (MOTIE, Korea), the Hallym University Medical Center Research Fund. The funder had no role in the design and conduct of the study, the collection, management, analysis, and interpretation of the data; the preparation, review, or approval of the manuscript; and the decision to submit the manuscript for publication.

## Conflict of interest

The authors declare that the research was conducted in the absence of any commercial or financial relationships that could be construed as a potential conflict of interest.

## Publisher’s note

All claims expressed in this article are solely those of the authors and do not necessarily represent those of their affiliated organizations, or those of the publisher, the editors and the reviewers. Any product that may be evaluated in this article, or claim that may be made by its manufacturer, is not guaranteed or endorsed by the publisher.

## Supplementary material

The Supplementary material for this article can be found online at: https://www.frontiersin.org/articles/10.3389/fnagi.2023.1196185/full#supplementary-material
