# Supplementary figures and images for: Association between migraine and Alzheimer’s disease: a nationwide cohort study
Source: Front Aging Neurosci. 2023 May 25;15:1196185. doi: 10.3389/fnagi.2023.1196185 (PMC10248237; doi:10.3389/fnagi.2023.1196185)

**
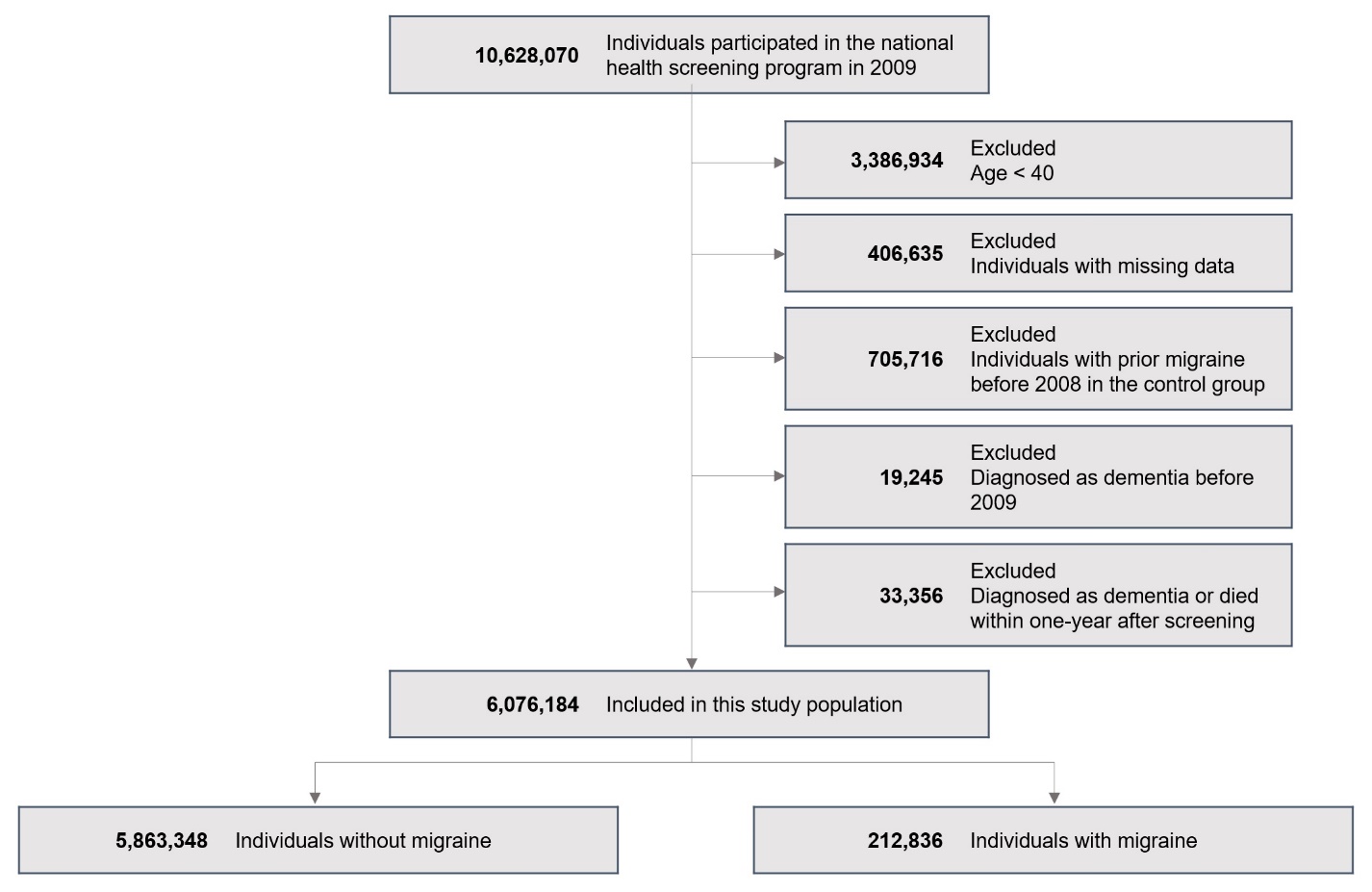
**

**Supplement Figure 1.** Flowchart of the study

Supplement: Supplementary file 1 [file Data_Sheet_1.docx]
